# Reconstructing cancer phylogenies using Pairtree, a clone tree reconstruction algorithm

**DOI:** 10.1016/j.xpro.2022.101706

**Published:** 2022-09-20

**Authors:** Ethan Kulman, Jeff Wintersinger, Quaid Morris

**Affiliations:** 1 Memorial Sloan Kettering Cancer Center, New York, NY, USA; 2 Deep Genomics, Toronto, ON, Canada

**Keywords:** Cancer, Genetics, Computer sciences

## Abstract

*Pairtree* is a clone tree reconstruction algorithm that uses somatic point mutations to build clone trees describing the evolutionary history of individual cancers. Using the Pairtree software package, we describe steps to preprocess somatic mutation data, cluster mutations into subclones, search for clone trees, and visualize clone trees. Pairtree builds clone trees using up to 100 samples from a single cancer with at least 30 subclonal populations.

For complete details on the use and execution of this protocol, please refer to Wintersinger et al. (2022).

## Highlights

• Pairtree is a fast and accurate clone tree reconstruction algorithm
• Pairtree builds accurate clone trees using up to 30 subclonal populations
• Pairtree provides tools to visualize and interpret clone trees

## Abstract (editor highlights)

Publisher’s note: Undertaking any experimental protocol requires adherence to local institutional guidelines for laboratory safety and ethics.

## Graphical abstract

**Figure:**
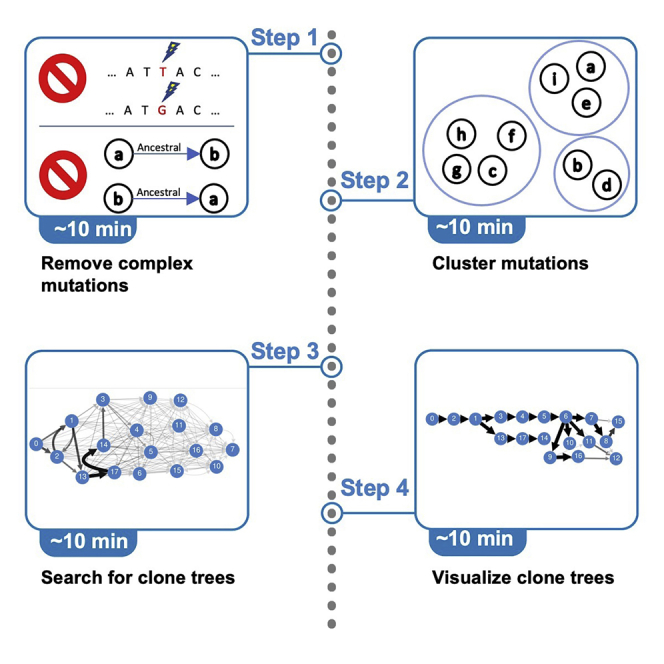

## Before you begin

Though many clone tree reconstruction tools exist, they differ in their intended purposes. Some methods are designed to build clone trees with limited numbers of subclones, such as PASTRI (Satas and Raphael, 2017), while others focus on building clone trees using longitudinal data, such as CALDER (Myers et al., 2019). As DNA sequencing has become cheaper, increasing numbers of cancer samples are sequenced to determine the mutational profiles that distinguish them from normal tissues, yielding large volumes of somatic mutation data. Pairtree differs from existing methods by accommodating these large amounts of data, such that it exploits information from many samples taken from the same cancer to build accurate clone trees even as the number of subclonal populations increases. Pairtree was benchmarked against many modern clone tree reconstruction methods, including PhyloWGS (Deshwar et al., 2015), CITUP (Malikic et al., 2015), PASTRI (Satas and Raphael, 2017), LICHeE (Popic et al., 2015), and CALDER (Myers et al., 2019). These results are detailed thoroughly in the Pairtree manuscript (Wintersinger et al., 2022) using both simulated and real data. Notably, Pairtree’s performance improved with more cancer samples while other methods worsened, allowing Pairtree to build clone trees that depicted cancer evolutionary histories with more detail than previously possible. Additionally, many other methods were unable to complete clone tree reconstructions in less than 24 h when given data containing 10 or more subclones.

This protocol details the specific steps for running Pairtree, as well as interpreting the clone trees produced by the method. Pairtree is available under the MIT license at https://github.com/morrislab/pairtree.

Before detailing how to use Pairtree, we provide an overview of the inputs it requires.

### Pairtree inputs

Most executables included with Pairtree operate on two files: (1) a simple somatic mutation(.ssm) file and (2) a parameters (.params.json) file.

We provide in the Pairtree repository the script vcf_to_ssm.py which can be used for converting data from a Variant Call Format (vcf) file to simple somatic mutation (ssm) file. The script vcf_to_ssm.py is an extension of source code provided in the file ssm_base_converter.py. The source code in ssm_base_converter.py can be extended by the user to translate any data format to an equivalent ssm file.

1. The .ssm file contains allele frequency data for point mutations occurring in one or more samples of a single cancer. These mutation calls can be derived from whole-genome sequencing (WGS), whole-exome sequencing (WES), or other targeted sequencing approaches. Each cancer sample (which we and others interchangeably refer to as “tissue samples”) contains a mixture of genetically heterogeneous cells. The resulting mutation data for each cancer sample comprises integer counts of the number of variant and reference reads at each mutation locus. The expected format of the .ssm file is detailed below, and an example .ssm file is shown in Figure 1.
  ***Note:*** Read count data should not be normalized, as Pairtree is designed to handle datasets with multiple cancer samples that vary in their read depth. Furthermore, Pairtree’s clone tree reconstructions benefit from including as many well-measured point mutations as possible, so long as they are not complex ones. Therefore, we recommend including both damaging and silent mutations.
  a. The .ssm file is a *tab-delimited* file, such that tabs separate each field within each line of the file.
  b. The first line of the .ssm file is the header. Each subsequent line provides data for an individual mutation. The .ssm file contains the following fields designated by the header:
    i. id: a unique ID assigned to each mutation. The required ID format is to assign the first mutation an ID of s0, and subsequent mutations s1, s2, ….. The s prefix denotes a simple somatic mutation, and the integer following it must be unique. The integers are recommended to be contiguous, but this is not enforced and permits deleting a mutation without needing to adjust subsequent IDs.
    ii. name: a text string for labeling mutations in the Pairtree output. The name field does not need to be unique, but it must be a text string that contains no white spaces. Common naming conventions include using the gene name, or the format <chromosome>_<position> (e.g., 1_12345, denoting a mutation occurring on chromosome 1 at locus 12345).
    iii. var_reads: a comma-separated vector of variant allele read counts at this mutation’s locus. Each element in the vector corresponds to the number of variant reads at this mutation’s locus in a specific tissue sample. The variant read counts must be non-negative integers, and the ordering of each tissue sample’s variant read counts must correspond to the ordering the tissue samples used in the .params.json file (see part (2) of this section below).
      **CRITICAL:** If the data only have one sample, a single value should be used for each mutation. If there are mutations that have only been called in a subset of the tissue samples, please refer to the section below on imputing and scaling read counts.
    iv. total_reads: a comma-separated vector of the total number of reads at this mutation’s locus. In other words, the total_reads for a mutation is the sum of the variant read count and the reference read count in a specific tissue sample. Each position in the array corresponds to the number of total reads at this mutation’s locus in a specific tissue sample. Total read counts must be whole numbers, and the ordering of each tissue sample’s total read counts must correspond to the ordering the tissue samples in the .params.json file.
      ***Note:*** Using both the var_reads and total_reads, a mutation’s variant allele frequency (VAF) can be computed as var_reads / total_reads. If there are mutations that have only been called in a subset of the tissue samples, please refer to the section below on imputing and scaling read counts.
    v. var_read_prob: a comma-separated vector of probabilities. Each position in the array corresponds to the probability of observing a read of the mutation’s variant allele for cells bearing the mutation in a specific tissue sample.
      **CRITICAL:** If the cells bearing the mutation are diploid and the mutation occurred on a chromosome with two copies, we would expect to observe the variant allele in half of our reads and would set the var_read_prob to 0.5. If the mutation occurred on a haploid chromosome (e.g., male sex chromosome), we would expect to always observe the variant allele and would set the var_read_prob to 1.0. If a copy-number aberration (CNA) occurred which duplicated the reference allele in the cell lineage bearing a particular mutation, then there will be two reference alleles and a single variant allele. In this case, we would expect to observe the variant allele 1 out of 3 times, and so we would set our var_read_prob to 0.3333. These are just a few examples of how the var_read_prob may want to be modified based on prior knowledge. Properly setting the var_read_prob is critical because Pairtree uses it to calculate the data-implied subclonal frequency as (var_reads / total_reads) / var_read_prob, representing the estimated proportion of cells bearing the mutation.

**Figure 1:**
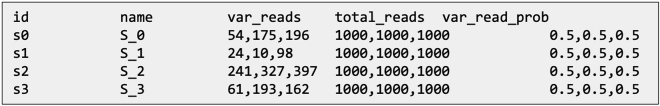
Example .ssm file
2. A parameter file (.params.json) provides Pairtree with information about the mutations listed in the .ssm file. The parameter file is specified in the JavaScript Object Notation (JSON) format, which is a text-based format for representing richly structured objects. The parameter file consists of three values stored using the following keys: samples, clusters*,* and garbage. The value for each key will be an array literal (commonly referred to as a list) that contains a specific type of data. These three keys and their associated values are described below, and an example .params.json file is shown in Figure 2.
  a. samples (required): a list of sample names. The order of the samples must match the order of entries for the var_reads, total_reads, and var_read_prob vectors specified in the .ssm file. The sample names can be any string value. Pairtree has been tested on datasets containing up to 100 samples, however, there is no restriction on the number of samples that can be input into Pairtree.
    ***Note:*** In general, Pairtree’s clone tree reconstructions benefit from more samples, although it’s important to consider when including more samples that Pairtree’s runtime and memory requirements increase linearly with the number of samples used.
  b. clusters (required): a list of lists specifying the mutations which belong to each subclone. Each sub-list contains the id values (which are specified in the .ssm file) for all mutations that belong to that cluster/subclone. All mutations belonging to a cluster are assumed to share the same subclonal frequency in a given tissue sample. Thus, we interpret the VAF of each individual mutation in a cluster as a noisy observation of its true subclonal frequency, after using the mutation’s var_read_prob to correct for ploidy.
    **CRITICAL:** Pairtree performs well on clone tree reconstruction problems with up to 30 subclones but performs poorly using 100 subclones. We recommend using no more than 30 subclones until improvements are made to Pairtree, as Pairtree’s runtime and memory requirements can increase super-linearly with larger numbers of subclones. Clustering mutations is described in further detail in the section Clustering mutations into subclones.
  c. garbage (optional): a list of mutation id values (id values are specified in the .ssm file) that will not be used by Pairtree when constructing clone trees. Including mutations in the garbage list excludes them from being used by Pairtree without removing them from the .ssm file. This may be useful if there is evidence that a mutation’s read count data is erroneous or contains a complex inheritance pattern (e.g., acquisition by an ancestral subclone followed by a loss in a descendent subclone). We call these mutations *garbage mutations* or *complex mutations* and describe how to identify them in the section titled identifying garbage mutations*.*

**Figure 2:**
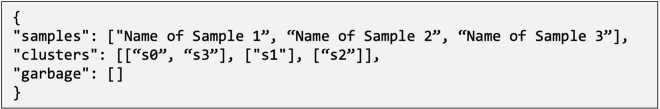
Example .params.json file

### How different inputs change the runtime and behavior of Pairtree

The following protocol was run using a standard modern laptop and uses a simulated dataset containing 3 samples and 30 subclones. Changing the number of samples or subclones will change Pairtree’s runtime. Generally, Pairtree’s runtimes increase super-linearly with the number of subclones and linearly with the number of samples. Because of this, we provide an extended review of how we would expect the runtimes and behavior of Pairtree to change when varying the input data. We have tested Pairtree with up to 100 subclones and 100 samples, though we do not recommend using more than 30 subclones as we expect Pairtree’s reconstruction accuracy to decrease with more subclones.

The table below provides the approximate ranges for the time required by Pairtree as a function of the number of subclones and samples. These approximate runtimes were derived from experiments using simulated data (see Figure S10 in the Supplementary Information in Wintersinger et al. (2022)). Note that the wall-clock runtime depends on the number of available CPUs because Pairtree will use multiple CPUs if available. For more details on the pairtree algorithm, please see the step-by-step method details section titled run pairtree*.*

### Pairtree expected wall-clock runtime table

**Table:** 

Number of subclones	Number of samples	Expected runtime (seconds)
3–10	up to 100	60–100
11–30	up to 100	100–300
31–100	up to 100	300–4,000
100+	up to 100	4,000+

### Imputing and scaling read counts

In a multi-sample setting, a particular mutation may not be called in all cancer samples. In this case, we must impute the mutation’s variant and total read counts in the samples where these data are missing. Usually, a mutation is not called because it is found at a very low frequency in the sample, or it is not present at all. This is deemed “missing not at random” (MNAR) because the fact that the data is missing suggests that the subclonal frequency of the variant is truly low. However, in some cases a mutation is not called because few reads, either variant or reference, mapped to its locus. In this case the variant is deemed “missing at random” (MAR), since we lack sufficient information about its true subclonal frequency.

In both the MAR and MNAR cases, we can set the mutation’s variant read count to 0. We will, however, want to set the total read count differently depending on whether we have sufficient information that it is MAR or MNAR. In a MAR case, e.g., it’s believed that no reads mapped to the locus, we can set the total read count to 1. A total read count of 1 indicates to Pairtree that we have no knowledge about the frequency of the variant in the sample, and this allows Pairtree to be more flexible when determining the tree-constrained subclonal frequency of the mutation. In the MNAR case, setting the total read count requires further care because it provides a soft constraint on the tree-constrained subclonal frequency that Pairtree can assign it. A low total read count permits Pairtree to have more flexibility when determining the tree-constrained subclonal frequency, while a high total read count permits less flexibility.

For example, observing 10 total reads and no variant reads would not be surprising if the subclonal frequency were 5%, but would be very surprising if instead 1,000 total reads were observed. For the MNAR case, there are a few common strategies for setting the total read count including (1) using the average total read count across samples for that locus, or (2) using the average total reads across all other loci in the sample.

### Reproducibility and quality control

It’s important that the quality of the input data be checked prior to running Pairtree, and that the results obtained using Pairtree are reproducible.

Quality control checks can be performed on the input data prior to constructing clone trees with Pairtree using the fix_bad_var_read_prob.py and removegarbage scripts included in the Pairtree GitHub repository. These scripts will check your input data for violations of the Infinite Sites Assumption (ISA), and for other complex mutation data that may cause Pairtree to construct poor clone trees. Further details on these scripts are provided in the step-by-step method details section.

This entire protocol can be run as a pipeline as shown in the bash script run_pipeline.sh that is provided in the Pairtree GitHub repository. This script can be copied and modified for use with other data and provides a template for reproducing results obtained using Pairtree.

### Software prerequisites and data requirements

Pairtree can be used on Linux or macOS. Pairtree and its accompanying utilities are all written in Python 3. The components included in the Pairtree source tree are detailed below along with their dependencies.

## Key resources table

**Table:** 

REAGENT or RESOURCE	SOURCE	IDENTIFIER
**Deposited data**		

methods.ssm (example input file)	Pairtree GitHub	https://github.com/morrislab/pairtree/blob/master/star_methods/methods.ssm
methods.params.json (example input file)	Pairtree GitHub	https://github.com/morrislab/pairtree/blob/master/star_methods/methods.params.json
run_pipeline.sh (example script for running the Pairtree utilities)	Pairtree GitHub	https://github.com/morrislab/pairtree/blob/master/star_methods/run_pipeline.sh

**Software and algorithms**		

removegarbage	Pairtree GitHub	https://github.com/morrislab/pairtree/blob/master/bin/removegarbage
clustervars	Pairtree GitHub	https://github.com/morrislab/pairtree/blob/master/bin/clustervars
pairtree	Pairtree GitHub	https://github.com/morrislab/pairtree/blob/master/bin/pairtree
plottree	Pairtree GitHub	https://github.com/morrislab/pairtree/blob/master/bin/plottree
summposterior	Pairtree GitHub	https://github.com/morrislab/pairtree/blob/master/bin/summposterior
fix_bad_var_read_prob.py	Pairtree GitHub	https://github.com/morrislab/pairtree/blob/master/util/fix_bad_var_read_prob.py
vcf_to_ssm.py	Pairtree GitHub	https://github.com/morrislab/pairtree/blob/master/convert_to_ssm/vcf_example/vcf_to_ssm.py
ssm_base_converter.py	Pairtree GitHub	https://github.com/morrislab/pairtree/blob/master/convert_to_ssm/ssm_base_converter.py
Git	Version control software	https://git-scm.com/
Projectppm	Perfect phylogeny model algorithm developed by Jia et al. (2018)	https://github.com/jwintersinger/projectppm
Numpy	Scientific computing package for creating and processing multi-dimensional arrays	https://numpy.org/
SciPy	Scientific computing package for solving mathematical problems	https://scipy.org/
scikit-learn	Machine learning package for performing classification, regression, and clustering	https://scikit-learn.org/stable/
Tqdm	Package for displaying progress of Python looping mechanisms	https://tqdm.github.io/
Numba	Optimization package to improve runtime of Python and NumPy functions	https://numba.pydata.org/
Colorlover	Python package containing a variety of color palettes	https://github.com/plotly/colorlover
Plotly	Graphing library for creating plots/figures	https://plotly.com/python/

## Materials and equipment

The experiments detailed during the protocol were performed using a MacBook Pro (2.3 GHz dual-core Intel Core i5, 8 GB of 2,133 MHz LPDDR3 onboard memory). This hardware is sufficient for the example data used in this protocol as it only contains 3 samples and 30 subclones. Larger datasets may benefit from more computing resources to finish in a timely fashion.

**CRITICAL:** The required memory and computational power necessary to run Pairtree will depend on the size of your data. The step-by-step method details and the troubleshooting sections have further information on how to reduce the run-times for the different executables in the Pairtree software package.

## Step-by-step method details

The following step-by-step method details sections describe how to run Pairtree and its utilities using a set of example files as inputs, comprising methods.ssm and methods.params.json. These data files are simulated data meant to demonstrate the use of our protocol. The same steps can be used to run Pairtree with other data.

**CRITICAL:** Your data are unique. The utilities included along with Pairtree can perform statistical checks and can flag potentially erroneous fields. These methods are inherently limited to identifying clear issues; more subtle errors may escape these automated checks.

### Clone our repository and install requirements

**Timing: < 10 min**

Prior to beginning our experiment, we need to clone the Pairtree repository and install dependencies. We describe in this section how to clone our repository using different package managers and install necessary dependencies.

1. Clone repository and install dependencies. Installation can be performed using mamba, conda, or pip.
  a. Clone the repository from GitHub and move into the Pairtree directory.

>git clonehttps://github.com/morrislab/pairtree

>cd pairtree

Then, you can install the dependencies using mamba, which is an accelerated version of conda.

>mamba create --name pairtree --file requirements.txt

>conda activate pairtree

Alternatively, you can install the dependencies using conda.

>conda install --list requirements.txt

>conda activate pairtree

A third alternative is to install the dependencies using pip.

>pip install -r requirements.txt

2. Download and build the modified *projectppm* library that fits subclonal frequencies to candidate clone trees. The following terminal commands assume you are currently in the */pairtree* directory.
  a. Navigate to */pairtree/lib* directory and clone the *projectppm* repository.
    >cd lib
    >git clonehttps://github.com/jwintersinger/projectppm
  b. Navigate to the */pairtree/lib/projectppm* directory and build the projectppm library.
    >cd projectppm
    >bash make.sh
    **CRITICAL:** Ensure that the *projectppm* repository is in the */pairtree/lib/* directory. The *pairtree* executable will look for the compiled *projectppm* in */pairtree/lib/projectppm*. If this step is not done properly, *pairtree* will not be able to use the *projection* algorithm to fit subclonal frequencies to the tree.

### Fixing incorrect ploidy

**Timing: < 5 min (when using data containing 3 samples and 30 subclones)**

Incorrect ploidy may occur when the wrong var_read_prob is assigned for a mutation in a given sample. These situations may arise due to uncalled loss of heterozygosity (LOH) or other missed copy number aberrations (CNAs). Uncalled LOH events may be simple to detect manually with a small dataset, however, verifying each var_read_prob in larger dataset may be more cumbersome. For uncalled LOH, we provide a script, fix_bad_var_read_prob.py, which attempts to detect uncalled LOH events and correct the var_read_prob.

3. Inspecting the methods.ssm file.
  a. When inspecting methods.ssm, we can see that the total read count (total_reads) across all mutations/samples is 1,000. We can also see that the var_read_prob is set to 0.5 across all mutations/samples, meaning we would expect to obtain a variant read about half of the time.
  b. For example, the mutation with the id of s14 has var_reads 269, 328, and 427 across the three samples resulting in VAFs of 269/1,000 = 0.269, 328/1,000 = 0.328, 427/1,000 = 0.427. With a var_read_prob of 0.5, we can then calculate the data-implied subclonal frequency for mutation s14 across the three samples as 0.269/0.5 = 0.538, 0.328/0.5 = 0.656, 0.427/0.5 = 0.854. These are noisy estimates for the percentage of cells in each sample bearing mutation s14. However, if we look at mutation s10 and perform the same calculations, we can see that in each of the 3 samples we have VAFs of 0.879, 0.698, 0.994. When calculating the data-implied subclonal frequencies for mutation s10, we obtain 0.879/0.5 = 1.758, 0.698/0.5 = 1.396, 0.994/0.5 = 1.988. These data-implied subclonal frequencies do not make sense, as we cannot have more than 100% of the cells in a sample bearing mutation s10. In this case, the data implies that we should have a var_read_prob of 1 across all samples for mutation s10, as there may have been an uncalled LOH event which resulted in the cells having only the variant allele. Although we can detect and correct this manually for our example data, we provide the fix_bad_var_read_prob.py script to perform this task automatically.
4. Properly configure the parameters for fix_bad_var_read_prob.py.
  a. There are a few notable command line arguments for the fix_bad_var_read_prob.py script that we may want to modify.
    i. In our case, we will use the –action argument which tells the script what to do if it finds a mutation that likely had an uncalled LOH event. We will pass the value of modify_var_read_prob to –action, which tells the program to update the var_read_prob value for any mutation that may have had an uncalled LOH or CNA event.
    ii. We can change the value that the var_read_prob gets updated to by passing a numeric value to the argument –var-read-prob-alt. The default value for –var-read-prob-alt is 1. We do not need to modify this value for our example.
    iii. If during the protocol some id values have been added to the list of garbage mutations and the user does not want them to appear in the results from running fix_bad_var_read_prob.py, then the flag –ignore-existing-garbage should be used to exclude these mutations from the analysis.

Please see the Pairtree GitHub for further information about the command line arguments for this script.

5. Run fix_bad_var_read_prob.py.
  a. We can now run the script using the following command.
    > python3 util/fix_bad_var_read_prob.py star_methods/methods.ssm star_methods/methods.params.json star_methods/methods.ssm star_methods/methods.params.json --action modify_var_read_prob
    The output from running this script should be as follows:
    num_bad_ssms=1
    bad_ssms=['s10']
    bad_samp_prop=0.033
    bad_ssm_prop=0.033
    We’ll briefly explain each line outputted by this script. The first line num_bad_ssms=1 states how many mutations from the .ssm file may have an incorrect var_read_prob. The second line bad_ssms=['s10'] lists the mutations that were found to have an incorrect var_read_prob. The third line bad_samp_prop=0.033 states the proportion of read count data that may have an incorrect var_read_prob. The fourth line bad_ssm_prop=0.033 states the proportion of mutations that have at least one sample with an incorrect var_read_prob.
  b. Finally, we can verify that the fix_bad_var_read_prob.py script adjusted the var_read_prob for s10 to 1 by checking the methods.ssm file. The line for mutation s10 should look as follows:
    s10 S_10 879,698,994 1000,1000,1000 1.0,1.0,1.0
    ***Optional:*** Create a directory to store your results from running fix_bad_var_read_prob.py. See the run_pipeline.sh script in the Pairtree source tree for an example of how to design a pipeline using multiple Pairtree components.

### Identifying garbage mutations

**Timing: < 5 min (when using data containing 3 samples and 30 subclones)**

Erroneous read count data can lead Pairtree to construct incorrect clone trees. Pairtree assumes a simple inheritance pattern for the mutations included in its clone trees. In most cases, the vast majority of point mutations in an individual cancer satisfy this simple pattern called the infinite sites assumption (ISA), such that each mutation occurs only once during the evolution of the cancer and does not revert to the reference allele (Singer et al., 2018). The Pairtree software package includes the removegarbage script to detect mutations with potential complex inheritance patterns where, e.g., they have a missed CNA call, suffered an ISA violation, or their variant read counts are corrupted with technical noise. We deem mutations affected thus as *garbage mutations*. We remove these garbage mutations because Pairtree cannot identify the correct evolutionary relationship they should have with respect to other mutations, so including them in the analysis can lead to incorrect trees. The removegarbage script provides an automated method for detecting and adding these mutations to the list of garbage mutations in the .params.json file.

6. Properly configure the parameters for removegarbage.
  a. There are several command-line arguments for removegarbage that may need to be set depending on prior knowledge and the data. In our example, we can use the default parameters.
  b. If there is reason to believe the data is particularly noisy, then the –prior parameter can be increased as it sets a prior probability on each pair of mutations having a garbage relationship. The default –prior is 0.2, reflecting a uniform prior with respect to the five possible pairwise relationship types (see Wintersinger et al. (2022) for more detail).
  c. If during the protocol some id values have been added to the list of garbage mutations and the user does not want them to appear in the results from running removegarbage, then the flag –ignore-existing-garbage should be used to exclude these mutations from the analysis.
7. Run removegarbage.
  a. We can run this script using the following command
    > python3 bin/removegarbage star_methods/methods.ssm star_methods/methods.params.json star_methods/methods.params.json
    The command may take a few seconds to run.
  b. The result of this script will be a modified garbage list in the methods.params.json. Specifically, the mutation with id s20 should have been placed into the garbage list, and the methods.params.json file should appear as follows:
    {
    “samples”: ["Sample 1″, “Sample 2″, “Sample 3"],
    “clusters”: [],
    “garbage”: ["s20"]
    }
    Mutation s20 is labeled as garbage because the data in each sample imply conflicting pairwise relationships with other mutations. In Sample 1, s20 has a high VAF, making it a likely candidate to be ancestral to most other mutations. In Sample 2, it has a similar VAF to most other mutations. In Sample 3, it has a very low VAF, making it likely to be a descendant of many other mutations. Because of these conflicting pairwise relationships, it’s likely that s20 is a garbage mutation.
    ***Note:*** This script may take much longer to run if the data used has many samples/and or mutations.

### Clustering mutations into subclones

**Timing: < 10 min (when using data containing 3 samples and 30 subclones)**

In order to run Pairtree, mutations must first be clustered into subclones in the .params.json file. There are several approaches for generating these clusters.

First, instead of building a clone tree, a *mutation tree* could be built. In this case, each mutation that appears in the .ssm file would be listed as a separate cluster in the .params.json file. The tree will then show the evolutionary relationships between mutations instead of between subclones. We recommend this approach if the data being used has 30 or fewer mutations.

Alternatively, the clustervars script can be used to generate the subclones. We will not go into detail on the different ways to configure the parameters for clustervars, as there are many possible configurations that require consideration. Please see the documentation in the Pairtree source tree for more information.

8. Run clustervars.
  a. We can run this script using the command.
    > python3 bin/clustervars star_methods/methods.ssm star_methods/methods.params.json star_methods/methods.params.json.
    This command may take up 30 s to run.
  b. The result of this script will be a modified clusters list in the methods.params.json. Specifically, the script will generate a list of lists containing four different clusters. The methods.params.json file should now appear as follows:
    {
    “clusters”: [["s10"], ["s2″, “s4″, “s5″, “s6″, “s8″, “s9″, “s11″, “s12″, “s13″, “s14″, “s19″, “s21″, “s22″, “s23″, “s24″, “s25″, “s27"], ["s0″, “s3″, “s7″, “s15″, “s16″, “s17″, “s28"], ["s1″, “s18″, “s26″, “s29"]], “garbage”: ["s20"],
    “samples”: ["Sample 1″, “Sample 2″, “Sample 3"]
    }
    After clustering mutations into subclones, it’s important to verify that these groupings make sense. Generally, these verifications involve ensuring that the VAFs are similar, although in some special cases they could be performed by ensuring that clusters do not contain mutually exclusive driver mutations. We can perform a manual check by looking at the groupings of mutations in each cluster in the methods.params.json file, then using the data in methods.ssm to verify that the mutations have VAFs which, when adjusted for ploidy, appear to reflect the same underlying subclonal frequency.
    The first cluster consists only of mutation s10, which is likely because there are no other mutations that had such high read counts across all 3 samples. The second cluster has quite a few different mutations s2, s4, s5, …. All these mutations have a var_read_prob of 0.5 and the same total_reads, so we can check whether their variant read counts are approximately the same to determine if they reflect the same subclonal frequency.
    All mutations in the second cluster are clearly present in all three samples, as their variant read counts are approximately 200 in the first sample, 300 in the second sample, and 400 in the third sample. We cannot necessarily use this information to conclude that all these mutations should definitely be placed in the same cluster i.e., two mutations with the same apparent subclonal frequency may not necessarily have arisen in the same cells, but may instead belong to distinct subpopulations with similar subclonal frequencies. Nevertheless, we have no reason to conclude that the mutations should be separated, given their similar read counts across all samples.
    The third cluster consists of mutations s0, s3, s7, ..., which all seem to have near-zero variant read counts in Sample 1 and are present in the other two samples. This is different than cluster 1 or cluster 2, both of which had non-zero variant read counts in all three samples. Finally, cluster 4 consists of mutations s1, s18, s26, and s29, all of which have near-zero variant read counts across all three samples.
    Although it’s difficult to say whether this clustering is correct by checking it manually, it does allow us to see that there are no obvious errors that will compromise Pairtree’s ability to construct clone trees.
    ***Note:*** Other non-Pairtree methods can be used to cluster mutation into subclones, such as PyClone (Roth et al., 2014), PyCloneVI (Gillis and Roth, 2020), and SciClone (Miller et al., 2014). If clustering is performed via another method, the results must be converted into the .params.json format Pairtree uses.

### Run pairtree

**Timing: < 10 min (when using data containing 3 samples and 30 subclones)**

All the work done in previous steps prepared our data for running the pairtree executable that builds clone trees. Properly configuring pairtree depends heavily on both prior knowledge and the data. This section will discuss some of the ways one can configure pairtree.

9. Properly configure the parameters for pairtree.
  a. In order to obtain the same exact output shown in this procedure when using the example data, we must pass the parameter –seed=5555 to pairtree. The argument passed to the seed parameter can be any non-negative integer less than or equal to 2^32^ – 1. The underlying randomness of the method used to search for clone trees will produce slightly different results on each execution of pairtree. Setting the seed parameter to the same value across multiple executions of pairtree will produce the same set of clone trees, assuming all other parameters and inputs are also held fixed.
  b. Besides setting the seed parameter, we will use the default parameters for pairtree when using the example data for this protocol. However, there are several other key parameters that could be modified when using different data.
  c. Pairtree offers two different methods for fitting subclonal frequencies to trees, termed projection and rprop.
    i. By default, pairtree uses the “Efficient Projection onto the Perfect Phylogeny Model” algorithm developed in Jia et al. (2018). This can be specified explicitly by passing –phi-fitter=projection to pairtree. This method maximizes the binomial likelihood for subclonal frequencies using a Gaussian approximation. It is fast to compute and produces fairly accurate results, making it generally a good choice for both small and large datasets. However, we find that sometimes the Gaussian approximation can lead to inaccurate results on larger datasets.
    ii. The alternative model is rprop, which is a gradient-based method that directly optimizes the binomial objective. This can be specified using the option –phi-fitter=rprop. This method typically produces more accurate results than projection but is more computationally intensive. By default, the rprop algorithm runs for 10,000 iterations. To manually set the number of iterations to some integer N, pass –phi-iterations=N to pairtree. If pairtree is producing poor results using projection, try switching to rprop. It is important to note that rprop may be computationally infeasible for more than 30 subclones, depending on how many cancer samples the data has and how powerful the available computing hardware is.
    iii. Running pairtree with the example data and parameters provided in this protocol will obtain the same exact results using either rprop or projection. This is because our example data is relatively simple and has had complex mutations withheld from the analysis (see the step-by-step method details section identifying garbage mutations*).* For data that is not so simple, it’s possible to obtain different sets of candidate clone trees when running pairtree using rprop versus projection. This can be particularly problematic when the data are well-fit by many possible clone tree configurations. The different results from the two algorithms may also be due to a poor approximation by the projection algorithm, or because rprop does not converge quickly enough.
  d. pairtree samples candidate clone trees using Markov Chain Monte Carlo (MCMC). To find high-quality trees, multiple MCMC chains are run, each of which sample a fixed number of trees. Two parameters related to MCMC can be modified to potentially improve results.
    i. By default, pairtree samples 3,000 trees with each MCMC chain. To set the number of trees sampled by each MCMC chain to some non-negative integer N, pass the argument –trees-per-chain=N to pairtree.
      ***Note:*** Pairtree’s runtime will scale linearly with the number of trees sampled by each MCMC chain.
    ii. Each MCMC chain discards a fraction of the total number of trees it samples at the beginning of its search to avoid including poor-quality trees in the results. By default, pairtree discards the first 1/3 of all trees sampled by each chain. This can be set manually to some proportion F (where F is between 0 and 1) by passing the argument –burnin=F to pairtree.
10. Run pairtree.
  a. We can run this script using the following command.
    > python3 bin/pairtree --params star_methods/methods.params.json star_methods/methods.ssm star_methods/methods.npz --seed=5555.
    ***Note:*** if the file methods.npz already exists and includes complete results from a previous execution of pairtree, then pairtree will halt so as to not overwrite these results.
  b. The result of this script is the creation of methods.npz, which is a zip archive. It includes the sampled clone tree structures, subclonal frequencies, tree likelihoods, and other data. This .npz file is then used in the next two steps to visualize the candidate trees.

### Running summposterior and interpreting the results

**Timing: < 10 min**

Now that we’ve run pairtree and generated an .npz file containing candidate trees, we can visualize these trees to determine which best fit our data. The tree representations shown here consists of nodes (or vertices), with arrows (or directed edges) connecting the nodes. An arrow starting at Node 0 and ending at Node 1 implies that Node 0 is the parent (i.e., the immediate ancestor) of Node 1 or equivalently that Node 1 is a child (i.e., an immediate descendant) of Node 0.

**Figure 3:**
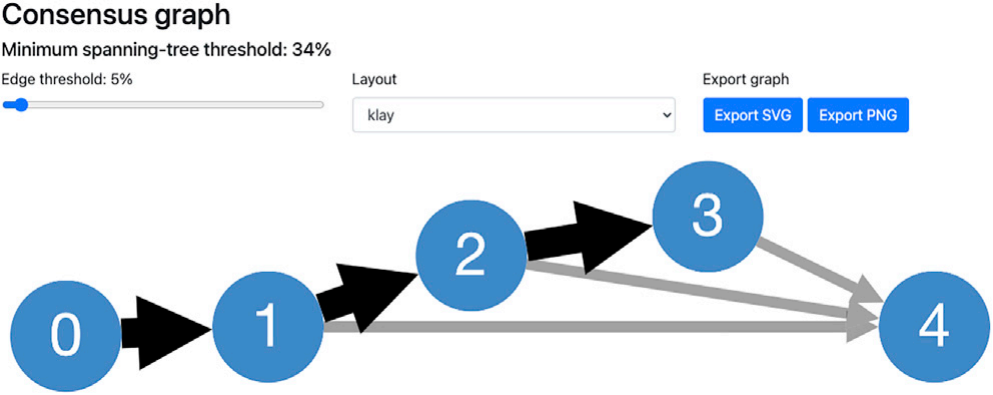
Consensus graph from methods.summposterior.html

**Figure 4:**
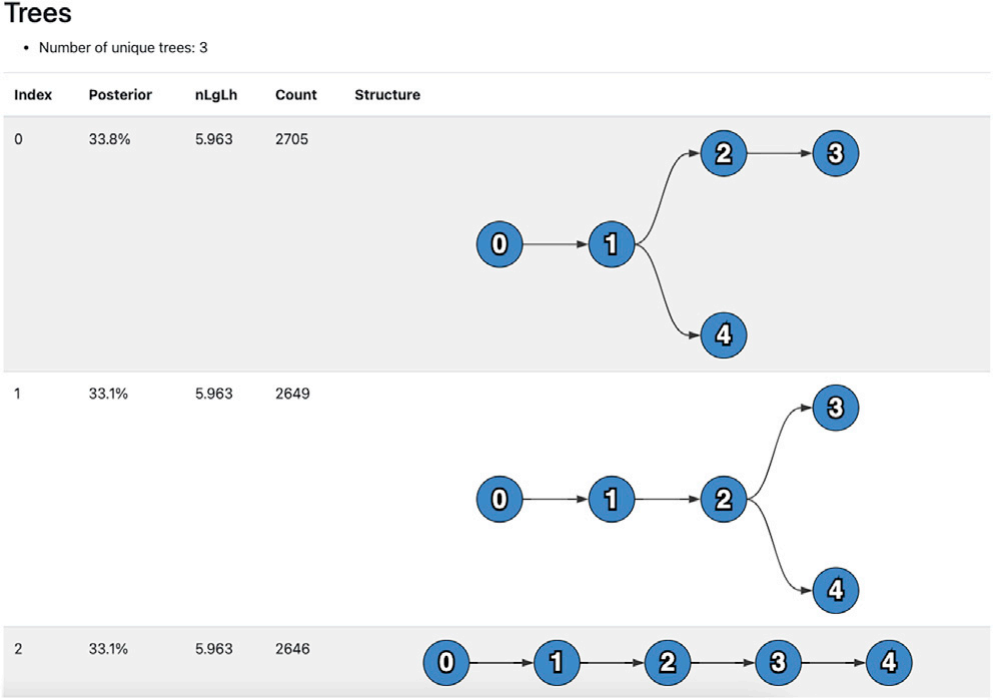
Trees from methods.summposterior.html

11. Properly configure the parameters for summposterior.
  a. The only recommended argument to alter for summposterior is –runid <run_name> where <run_name> is some string that describes the experiment. This label will be used within the HTML results file.
12. Run summposterior.
  a. We can run this script using the follow command.
    > python3 bin/summposterior --runid methods star_methods/methods.ssm star_methods/methods.params.json star_methods/methods.npz star_methods/methods.summposterior.html
  b. This script generates the methods.summposterior.html file, which contains a list of candidate trees and some basic information about them.
  c. Please note that if pairtree is run without setting the seed parameter to the exact value we did in the previous section titled run pairtree, then the file obtained after running summposterior may not match the images shown here.
13. Interpreting the results from summposterior.
  a. The summposterior file has three sections that we will first summarize. Then, we will discuss the results we obtained for each of these sections after running summposterior with the example data.
    i. Consensus Graph: provides an overview of the distributions of evolutionary relationships between subclones across all sampled trees. In a tree, each subclone only has one parent; in the consensus graph, subclones can have multiple parents corresponding to the parents of that subclone in at least one of the trees being summarized. This graph shows the user the full spectrum of possible relationships that are supported by the data.
      Each edge, i.e., parent-child relationship, between subclones is assigned a probability weight according to the data likelihoods of the trees in which that edge was present. This weight can be interpreted as the probability that a head node is the parent of the tail node, as such the weights of the incoming edges to a subclone sum to one. By changing the interactive *Edge threshold*, the user can filter out less certain relationships to show only those most supported by the data. The minimum spanning-tree threshold is the minimum value across the nodes of the maximum parent probability for that node; in other words, raising the edge threshold above this value would result in the graph becoming disconnected.
    ii. Trees: provides an overview of the most likely candidate trees, ordered by descending posterior probability. This section is detailed below.
      In the Trees section of the summposterior output, the top candidate trees are rendered. As shown in Figure 4, the methods.summposterior.html file will have three candidate trees, each with different branching patterns. The Posterior column lists the posterior probability for each tree. The nLgLh column lists the normalized negative log-likelihood for each tree. The Count column lists the number of times each tree was found during MCMC. The Structure column shows a visualization of the candidate clone tree.
      For each tree shown in the methods.summposterior.html file, pairtree computed the same negative log-likelihood (*nLgLh*), indicating that each is equally supported by the data. The *nLgLh* is normalized relative to the number of cancer samples and mutations, effectively indicating the likelihood “cost” in bits per mutation per sample that the tree incurred. Lower values reflect trees that better fit the data.
      Since the hardware this procedure was run on has 4 cores, each core had an MCMC chain that sampled 3,000 trees, which gives us a total of 12,000 trees. Since our –burnin when running pairtree was set to the default of 1/3, there were a total of 12,000/3 = 4,000 samples discarded. Therefore, the sum of all the rows for Count should add up to 8,000 (since 12,000–4,000 = 8,000).
      In the Structure column, we can see the visualizations for each of our three candidate clone trees. All trees have as their root Node 0, representing the non-cancerous population that is ancestral to all cancer populations. Each subsequent tree node represents a cancerous subclone whose mutations are given by the clusters specified in the .params.json file. For example, in our methods.params.json file, the first cluster contains only the s10 mutation, and corresponds to Node 1 in every candidate tree.
      Observe that the mutation clusters are constant across all sampled trees, but the tree structures and subclonal frequencies (and thus the computed data likelihoods) differ. The next section titled running plottree and interpreting the results focuses on interpreting the first tree, listed with tree index 0.
      ***Note:*** Often, multiple clone trees fit the data equally well. In these cases, the choice of clone trees could be based on assumptions about the dynamics of tumor evolution (Sottoriva et al., 2011), metastatic spread (El-Kebir et al., 2018), or spatial spread. Though, in general, we recommend examining the consensus graph (Figure 3) to distinguish parts of the clone tree that are well-supported by the data and those that are unconstrained by the data; and potentially collecting data from more samples to resolve uncertainties.
    iii. Diversity indices: shows three different measures that depict how heterogeneous the subclonal composition of each cancer sample is. For further information, please refer to the Pairtree documentation.

### Running plottree and interpreting the results

**Timing: < 10 min**

Now that we’ve run pairtree and generated an .npz file containing candidate trees, we will visualize and interpret the results for the candidate clone tree with the highest posterior probability.

**Figure 5:**
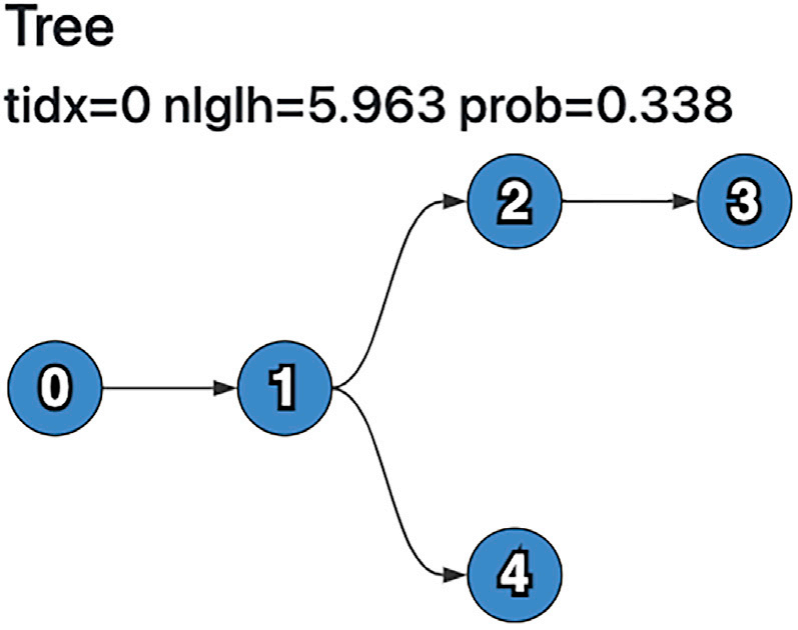
Candidate tree from methods.plottree.html

14. Configure the parameters for plottree.
  a. There are two parameters for the plottree script that are often set by the user.
    i. The file produced by plottree can be labeled by passing the argument –runid=<run_name>, where <run_name> is some string that describes the experiment and the selected clone tree.
    ii. A specific tree shown in the summposterior file can be plotted by passing the argument –tree-index=N, where N is the tree index. By default, plottree uses index 0, corresponding to the tree with the highest posterior probability. Examining other trees, as well as the consensus graph from summposterior, can illustrate plausible alternative trees that should be considered before making conclusions about the data.
15. Run plottree.
  a. We can run this script using the follow command.
    > python3 bin/plottree --runid methods star_methods/methods.ssm star_methods/methods.params.json star_methods/methods.npz star_methods/methods.plottree.html
  b. This script generates the methods.plottree.html file, which contains detailed information about a specific tree and how the input data relates to it.
16. Interpreting the results from plottree.
  a. Interpreting trees can be difficult because of the wealth of information that accompanies them. We will illustrate how to interpret one candidate tree found by pairtree during this procedure.
  b. The candidate tree we will interpret is shown in Figure 5*,* which was assigned index 0 in the methods.summposterior.html file output after running summposterior. We will analyze the placement of each subclone in this candidate tree, and use information from the tables in the methods.plottree.html file to understand why pairtree found this structure.
    i. Node 0 represents the non-cancerous cell population from which all cancerous subclones descended, as described in the previous section running summposterior and interpreting the results*.*
    ii. Node 1 represents the cluster containing only mutation s10. During this protocol, we corrected this mutation’s var_read_prob to 1.0, as its read counts suggested it originated from a haploid locus. The corrected var_read_prob still results in high estimated subclonal frequencies (near 1.0) for this subclone across all samples. Since the estimated subclonal frequencies of the other subclones (represented by Nodes 2–4) are much lower, Node 1 cannot be positioned such that it is a descendant of anything but the non-cancerous population (Node 0). Therefore, Pairtree’s placement of Node 1 as ancestral to all other subclones in the tree is reasonable.
    iii. Node 2 corresponds to a mutation cluster whose read counts yield data-implied subclonal frequencies well above zero across all three samples. Because these data-implied subclonal frequencies across all three samples are approximately 0.5, this node is unlikely to be a descendent of either Node 3 or Node 4, which are both absent from one or more samples.
      Suppose we placed Node 2 as a descendant of Node 3. The data-implied subclonal frequency for Node 2 in Sample 1 is 0.47, while the data-implied subclonal frequency for Node 3 in Sample 1 is 0.10. The tree constraints implied by the Infinite Sites Assumption require that each subclone’s frequency is at least as high as the sum of all its children’s frequencies across each sample, given that the mutations of a subclone are inherited by all its children. This would imply that the true subclonal frequency of Node 3 in Sample 1 must be at least 0.47, resulting in a severe mismatch relative to the 0.10 subclonal frequency implied by the data before considering tree structure constraints. As a result, either Node 3’s frequency would have to be much higher than implied by the data, or Node 2’s frequency would have to be much lower. Either case would incur a large amount of error between our data implied subclonal frequencies, and the tree-constrained subclonal frequencies. Moreover, we would have to adjust the tree-constrained subclonal frequency similarly for Sample 2 and Sample 3. To avoid this, we can place Node 2 in the tree such that it’s ancestral to Node 3 and Node 4, which does not impose any deviation from the data-implied frequencies. Consequently, it is reasonable for pairtree to prefer this relationship.
    iv. Node 3 represents the cluster containing mutations whose read counts suggest they are absent from Sample 1, but present in Sample 2 and Sample 3. Although this subclone is present in Sample 2 and Sample 3, its data-implied subclonal frequencies in these samples are around 0.4, making it less prevalent than Node 1 and Node 2. Therefore, it is plausible that Node 3 is a descendant of both Node 1 and Node 2.
    v. Node 4 represents the cluster containing mutations whose data-implied subclonal frequencies are near zero in all three samples. Pairtree placed it on another branch relative to Node 2 and Node 3, making it a direct descendant of Node 1. The placement of this node demonstrates the difficulties of deciding which candidate tree is likely to be correct. Placing this node on a separate branch incurs no error, but neither does making a linear branching pattern (see Figure 4, tree with index 2). Similarly, there is zero error incurred when Node 4 is a direct descendant of Node 2 (see Figure 4, tree with index 1).
      ***Note:*** With more cancer samples, pairtree can better resolve ambiguities like this. Without providing more samples, it’s possible to use other knowledge (such as a prior belief about how common branching vs. linear relationships are in a particular cancer type) to favor one tree over another, even if they have similar posterior probabilities.
  c. To evaluate the error rate between the tree-constrained subclonal frequencies and the data-implied subclonal frequencies, refer to the *Interleaved subclonal frequencies* table in the HTML file produced by plottree. Consider each triplet of rows. The first shows the tree-constrained frequencies fit to the tree; the second shows the data-implied frequencies, independent of any tree constraints; and the third shows the absolute difference between these, termed the tree error.
  d. To inspect the data-implied frequencies for each mutation within the mutation clusters, refer to the *VAFs (corrected)* table in the HTML file outputted by plottree.
  e. Although the trees depicted here are simple, the procedure we used to evaluate and interpret them can be applied to more complex trees constructed by pairtree.
    ***Note:*** For further information on the tables in the HTML file produced by plottree, please refer to the Pairtree documentation.

## Expected outcomes

The expected outcome of this protocol is a zipped archive file describing a set of candidate clone trees (see the step-by-step method details section titled Run pairtree). The clone trees and their related data can be visualized and interpreted using the summposterior and plottree software programs. Figures 3, 4, and 5 show some of the results obtained from running this protocol using a set of example data.

## Limitations

Pairtree uses the somatic mutation data and cluster assignments provided by the user to construct clone trees. If this data is particularly noisy or the mutation cluster assignments are poor (with mutations of substantially different implied frequencies placed in the same cluster), Pairtree will not be able to construct accurate trees. Furthermore, Pairtree expects mutations to obey the Infinite Sites Assumption (ISA). We provide utilities that attempt to remove mutations whose data are unduly noisy or that violate the ISA, but these tools may miss subtle cases that will nevertheless compromise the resulting trees. Note that Pairtree is not appropriate if one expects that the ISA is frequently violated (e.g., on more than 20% of mutations).

In some cases, multiple trees are consistent with the input data. Pairtree can only identify the parts of the clone tree constrained by the data and will not necessarily identify a single best fitting tree. In these cases, either additional data must be collected, or additional assumptions must be made to identify a single best fitting tree.

Pairtree is agnostic to classification of mutations (e.g., damaging vs. benign or driver vs. passenger) and cancer type when performing reconstructions. These classifications may be useful for resolving ambiguities and interpreting candidate clone trees.

Explainability and interpretability of trees is a difficult problem that is dependent on how much prior knowledge one has about the data and the cancer that generated it. The example we provide here is not comprehensive and is meant only to demonstrate the process of interpreting a tree without drawing on prior knowledge about the data.

## Troubleshooting

### Problem 1

I am getting the following error when trying to run *pairtree*:

Exception occurred in child process: dlopen …/pairtree/bin/../lib/projectppm/bin/libprojectppm.so, 0×0006): tried: '…/pairtree/bin/../lib/projectppm/bin/libprojectppm.so’ (no such file), '/usr/lib/libprojectppm.so’ (no such file).

### Potential solution

The projectppm repository has not been cloned into the */pairtree/lib* directory or its binaries have not been compiled. Please refer to our step-by-step method details section titled clone our repository and install requirements. Step (2) of this guide details how to clone and build projectppm.

### Problem 2

I have large amounts of data missing from some samples and/or mutations. How should I prepare my data for Pairtree?

### Potential solution

Curating your data prior to using Pairtree is highly recommended. First, we suggest removing samples or mutations with a lot of missing data, at least initially. Mutations that are uncalled in many samples that you do believe are MAR should be placed in the list of garbage mutations in the .params.json file. Additionally, samples with a lot of MAR data, or for which you believe most measurements are unreliable should be excluded from your inputs to Pairtree. In such cases of missing or noisy data, try building trees with only the subset of data you trust most. You can then gradually add less reliable mutations and/or samples to see how they change the resulting trees. For an alternative strategy that does not require discarding data, please refer to the section of this protocol titled imputing and scaling read counts.

### Problem 3

Pairtree’s mutation clustering method (clustervars) is producing poor results.

### Potential solution

First, try tweaking the hyperparameters used by the clustering algorithm (see the clustervars documentation). Alternatively, try using a different clustering algorithm such as PyClone or SciClone (as previously mentioned in the step-by-step method details section titled clustering mutations into subclones). This will require converting the output of these methods to the format used in Pairtree’s .params.json file.

### Problem 4

Pairtree’s mutation clustering method is too slow. How can I reduce the runtime of clustervars?

### Potential solution

There are two ways to decrease the runtime for clustervars. First, try changing the clustering model to linfreq using the argument –model=linfreq. This clustering method does not require computing pairwise relationships between individual mutations before clustering can begin and so is often much faster. Second, reduce the number of iterations for each Gibbs sampling chain by setting –iterations=N, where N is some non-negative integer. The default for N is 1,000.

### Problem 5

The candidate trees Pairtree produces have high nLgLh, indicating a mismatch between the subclonal frequencies fit to the tree and those implied by the data. How can I improve the result?

### Potential solution

First, if you are building clone trees rather than mutations trees, ensure the mutation clusters appear reasonable. Use the plotvars script to inspect the data-implied frequencies of the mutations in each cluster, and verify that any two mutations placed in the same cluster have similar frequencies across samples. To improve the clusters, refer to strategies listed in problem 3. Next, try increasing the –trees-per-chain parameter when running pairtree to 5,000 or 10,000. It will take longer for pairtree to complete, but this should provide a better approximation of the true posterior. Finally, try using the rprop method to fit subclonal frequencies instead of projection (for more information see the step-by-step method details section titled run pairtree).

## Resource availability

### Lead contact

Further information and requests for resources should be directed to the lead contact Dr. Quaid Morris (morrisq@mskcc.org) or Ethan Kulman (kulmane@mskcc.org).

### Materials availability

This study did not generate any new materials.

## Data Availability

Pairtree’s source code is available under the MIT license at https://github.com/morrislab/pairtree/. The version of record used to run this protocol: https://zenodo.org/badge/latestdoi/140898706.
